# A Comparison of Human Neutrophils Acquired from Four Experimental Models of Inflammation

**DOI:** 10.1371/journal.pone.0165502

**Published:** 2016-10-25

**Authors:** Alexander A. Maini, Marc J. George, Madhur P. Motwani, Richard M. Day, Derek W. Gilroy, Alastair J. O’Brien

**Affiliations:** 1 Centre for Clinical Pharmacology, Division of Medicine, University College London, London, United Kingdom; 2 UCL Applied Biomechanical Engineering Group, Division of Medicine, University College London, London, United Kingdom; University of Calgary, CANADA

## Abstract

Defects in neutrophil function have been implicated in a wide spectrum of clinical conditions. Several models are employed to study activated human neutrophils akin to those found at a site of inflammation. These include whole blood (WB) *ex vivo* stimulation with lipopolysaccharide (LPS) and *in vivo* techniques: cantharidin blister, skin windows and intra-dermal injection of UV-killed *E*.*coli* (UVKEc). Neutrophils obtained from these have never been compared. We compared the activation status of neutrophils from each technique in order to inform the optimal model for use in human studies. Healthy male volunteers were randomised to undergo one of the four techniques (n = 5/group). LPS: WB stimulated with 1ng/ml of LPS for 4 hours. Cantharidin: 12.5μl of 0.1% cantharidin elicited a single blister, aspirated at 24 hours. Skin windows: four 6mm mechanical-suction blisters created, de-roofed and an exudate-collection chamber placed over the windows for 4 hours before aspiration. UVKEc: 1.5 x 10^7^ UVKEc injected intra-dermally. A single 10mm mechanical-suction blister formed and aspirated at 4 hours. Unstimulated WB used as the control. Flow cytometry was used to determine activation status using CD16, CD11b, CD54, CD62L and CD88. Functional status was assessed with a phagocytosis assay. The pattern of neutrophil activation was similar in all models. Neutrophil CD11b was elevated in all models, most markedly in UVKEc (p<0.0001), and CD54 was also elevated but only significant in the LPS model (p = 0.001). CD62L was significantly reduced in all 4 models (p<0.0001) and CD88 was also suppressed in all. There were no changes in CD16 in any model, neither was there any significant difference in the phagocytic capacity of the neutrophils. In summary, there are no significant differences in activation marker expression or phagocytic capacity in the neutrophils obtained from each technique. Therefore we believe whole blood stimulation is the best model in experimentally challenging inpatient populations.

## Introduction

Neutrophils play a vital role in the innate immune system, the human body’s first line of defence from infection and tissue injury. Defects in neutrophil function have been implicated in a wide range of diseases, from congenital syndromes such as Chronic Granulomatous Disease [1] through to acquired defects in an array of conditions including Chronic Obstructive Pulmonary Disease [2], Crohn’s disease [3], Sepsis [4], Acute Alcoholic Hepatitis [5], and Cirrhotic Liver Disease [6].

Traditionally, human neutrophil function has been investigated *ex vivo* following isolation from fresh blood. This allows for large numbers of cells to be harvested and a wide array of phenotypic and functional studies to be performed for each study participant. However, circulating neutrophils have been shown to have significantly different characteristics to those found at inflammatory sites or organs. For example, compared to circulating, inflammatory site neutrophils have altered expression of cell-surface proteins including shedding of L-selectin (CD62L) and up-regulation of the constitutively-expressed integrins: LFA1 (β2 integrin CD11a complexed with CD18) and Mac-1 (CD11b complexed with CD18) [7]. These changes result in ‘priming’ of neutrophils, enhancing their key functions such as phagocytosis and oxidative burst to improve their bactericidal ability [8,9].

Thus, obtaining primed and steady state neutrophils is vital when studying their role in health and disease. A common and straight-forward method of achieving this is to stimulate whole-blood (WB) *ex vivo* with lipopolysaccharide (LPS)[10]. However, neutrophils stimulated in this manner have not been recruited to a site of inflammation from the circulation and have therefore not undergone the active process of tethering, rolling, adherence, and trans-migration through the endothelium [7].

In the age of translational biomedical research, human models that allow the study of neutrophils obtained from an environment more akin to an inflamed extravascular compartment are employed. Such models include the cantharidin blister [11,12] skin windows (generated by de-roofing blisters formed by negative suction) [9,13,14] and most recently described, intra-dermal (i.d.) injection of UV-killed *E*. *coli* (UVKEc) and subsequent blister formation over the site of injection [15]. While these models provide a way to acquire neutrophils that best represent this activated state, they do come with a cost in terms of acceptability to volunteers.

It has not been shown whether the primed neutrophils obtained from each of these three dermal models differ significantly from those primed by *ex vivo* stimulation with LPS, or indeed from each other. This information would aid selection of the most appropriate method for obtaining primed neutrophils for human studies.

In this paper we present data comparing the activation state of neutrophils obtained from the three dermal models of acute inflammation compared with WB stimulation with LPS. Employing flow cytometric analysis we demonstrate that the pattern of neutrophil cell-surface activation markers is similar across all four models and that there is no significant difference in the phagocytic capacity of the neutrophils obtained. Given the additional complexity of performing the dermal models relative to WB stimulation with LPS, we discuss the advantages and disadvantages of each model in detail.

## Materials and Methods

Young, healthy, non-smoking male volunteers between the ages of 18–45 were recruited. Exclusion criteria included any history of chronic illness, allergies, recent acute illness (<1month), vaccination within the past 3 months and regular medication (prescribed, over-the-counter or recreational). Some volunteers were used to optimise the models used (n = 7) and subsequent volunteers were randomly assigned to undergo one of the four experimental techniques (n = 5/group).

All three dermal models were performed on the ventral aspect of the non-dominant arm after shaving and disinfecting the skin, using an aseptic technique.

The study was approved by University College London (UCL) Institutional Ethics Committee (Project IDs: 4602/002, 5051/001, 5061/001). Informed written consent was taken from all the volunteers. All procedures were in accordance with the Helsinki Declaration of 1975, as revised in 1983.

### I.d. injection of UVKEc and blister formation

The technique for i.d. injection of UVKEc and subsequent inflammatory exudate collection via blister formation has recently been described [15]. Briefly, 1.5 x 10^7^ UVKEc in 100 μl saline were injected i.d. After 4 hours, a single 10mm blister was raised over the injection site by a suction chamber connected by tubing to a negative pressure instrument (NP-4, Electronic diversities Ltd., MD, USA). After securing on the chamber, negative pressure was gradually increased from 4 to 6–7 inches of mercury and kept at 37°C until the blisters had formed (approximately 60–90 minutes). The blister was aspirated and the exudate centrifuged at 500g for 5 minutes at 10°C. The cell pellet was resuspended in buffer (PBS (Gibco, Grand Island, NY) + 5mmol/l glucose + 0.2% BSA (Sigma, UK)) and kept on ice before being processed. The 4-hour time-point was selected as this has been shown to represent the peak of neutrophil influx and TNFα production within the model [15].

### Cantharidin Blister

The technique for inducing the cantharidin skin blister has previously been described [12]. Briefly, a single blister was elicited by applying 12.5 μl of 0.1% cantharidin (Cantharone, Dormer Laboratories). The blister was aspirated at 24 hours and the exudate centrifuged at 500g for 5 minutes at 10°C. The cell pellet was resuspended in buffer and kept on ice before being processed.

### Skin Windows and Exudate Collecting Chamber (ECC)

**Fig 1:**
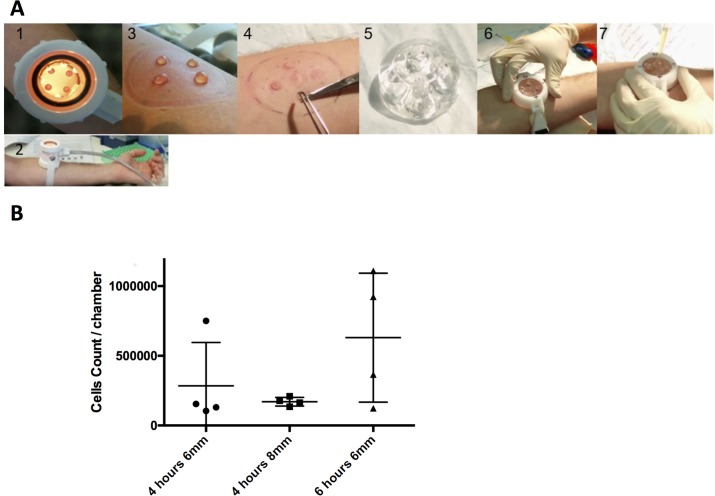
The Exudate Collecting Chamber (ECC) Model. (A) The technique employed in the ECC Model: 1&2: Blisters were formed by mechanical suction. 3: Formed blisters. 4: Blisters de-roofed to create skin windows. 5: Silicon ‘Exudate Collecting Chamber’. 6: ECC positioned over skin windows and kept in place by specially designed strap. 7: 50% serum introduced into chamber and then sealed with plugs. (B) Cell counts per ECC chamber achieved with different conditions during model optimisation (50% autologous serum used throughout). Mean ± SD displayed.

We employed a modified version of the skin-window technique previously described [13] **(Fig 1A).** This was optimised by investigating the effect of modifying the key variables **(Fig 1B).** We found that 50% serum encouraged neutrophil migration but that PBS, plasma and heat-treated serum did not (cell-count <10,000 cells in each). We trialled two time-points: 4 and 6 hours. Although it appeared that more cells were present at 6 hours, it was less well tolerated by subjects and the cell numbers at 4 hours were sufficient for the assays we employed. 8mm blisters did not confer any benefit over 6mm blisters. Therefore, the 6mm blister and 4-hour time-point were selected for use in the study protocol.

In the final protocol, four 6mm diameter blisters were simultaneously raised by applying a suction chamber as described above. After the blisters had formed they were de-roofed with sterile scissors. A novel one-use silicon ECC (MED-6015 Optically Clear Low Consistency Silicone Elastomer from Polymer Systems Technology Ltd, UK) manufactured from a 3D-printed mould was fitted over the four skin windows and held in-place by a bespoke strap. The chambers were each injected with 1ml of 50% autologous serum in PBS and left in place for 4 hours before being removed and the exudate aspirated. The serum from the 4 chambers was pooled and centrifuged at 500g for 5 minutes at 10°C. The cell pellet was resuspended in buffer and kept on ice before being processed.

### Fresh Whole Blood (WB)

Fresh, unstimulated WB was used as the control for all studies. Venous WB was collected in lithium heparin containing tubes (LH, Grenier Bio-One Vacuette). 1ml was lysed with 10ml ACK lysis buffer before being centrifuged at 500g for 5 minutes at 10°C. The cell pellet was resuspended in buffer and kept on ice before being processed.

### WB stimulation with LPS

WB stimulation with LPS has previously been described [16]. Briefly, venous WB was collected in lithium heparin containing tubes prior to stimulation. 1ml of WB was diluted 1:5 in RPMI (Gibco, Grand Island, NY) in 15ml polypropylene tubes (Fisher Scientific, Grand Island, NY) and stimulated with 1ng/ml of LPS (*Salmonella abortus equi* S-form [TLR*grade*], Enzo Life Sciences, Farmingdale, NY), within 30 minutes of draw, for 4 hours (37°C, 250rpm). After incubation, samples were centrifuged at 2000g for 10 min at 20°C. The supernatant was removed and the remaining pellet lysed with 2ml ACK lysis buffer (Lonza, Basel, Switzerland) prior to centrifugation at 500g for 5 minutes at 10°C. The cell pellet was resuspended in buffer and kept on ice before being processed.

Blood samples treated in an identical manner, without the addition of LPS, were used as an additional paired-control for these studies (4-Hr WB).

### Numerating cells

Cells from the three dermal blister models were enumerated using a haemocytometer with trypan blue exclusion. Cells from ACK-lysed whole blood were enumerated using the Countess Automated Cell Counter (Invitrogen).

### Flow cytometric analysis of lineage and cell-surface markers

Flow cytometry was used to determine the activation state of neutrophils from WB or the dermal models. This panel was based on activation markers previously used for neutrophils in bronchoalveolar lavage (BAL) fluid [17]. Cells were incubated with Human Trustain FcX (Fc Receptor Blocking Solution) (Biolegend, UK) for the prevention of non-specific antibody binding and were stained for cellular activation using anti-human CD16 (APC), CD11b (PerCP-Cy5.5), CD54 (FITC), CD62L (BV421) and CD88 (PE/Cy7) (all Biolegend, UK).

**Fig 2:**
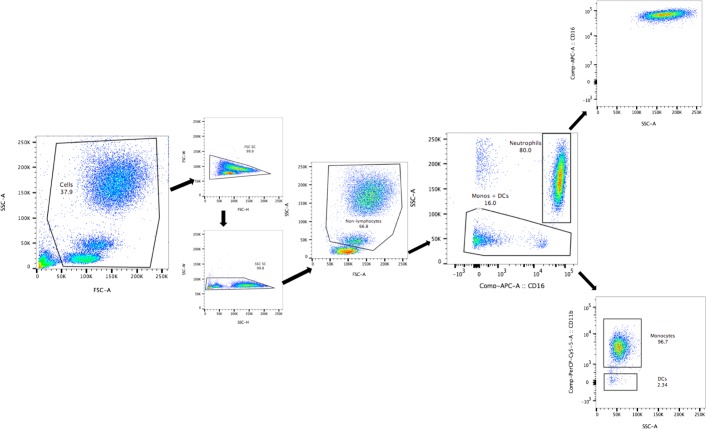
Flow Cytometry Gating Strategy Representative dot plots of flow cytometry gating strategy for activation markers (FSC, SSC, CD16-APC, CD11b-PerCPCy5.5, CD88-PECy7, CD54-FITC, CD62L-BV421). Exclusion of debris followed by exclusion of doublets. Exclusion of lymphocytes on FSC/SSC. Division of remaining cells by SSC and CD16 into neutrophils (SSC^inter-hi^CD16^hi^) and mononuclear cells (SSC^lo^CD16^lo-hi^). Mononuclear cells were then further subdivided by CD11b into monocytes (SSC^lo^CD11b^hi^) and dendritic cells (DCs) (SSC^lo^CD11b^lo^). Sample shown using human whole blood.

Cells were analysed using LSR Fortessa (BD Biosciences) with 4 lasers (Violet (405nm), Blue (488), Green (561nm), Red (640nm)). Gating for data acquisition was performed using BD FACSDiva 8.0.1. Flow cytometry data were analysed using FlowJo v10.0.7 (Tree Star, Inc.). Gating strategy is described in **Fig 2**. Results are expressed as a percentage of single cells and/or as geometric mean fluorescence intensity (MFI).

### Phagocytosis assay

Phagocytosis assays utilising flurochrome-labelled BioParticles have been previously described [19] however we employed a modified technique, which allowed substantially smaller numbers of cells to be used.

Approximately 1x10^7^ (33μg)/well *Escherichia coli* BioParticlues conjugated to Alexa Flour 488 (Molecular probes, Inc. Eugene, OR) were plated in 96 well plates (Costar, Corning International, Corning NY) and were opsonised with 50% autologous serum for 30 minutes at 37°C. After 30 minutes they were centrifuged at 3000g for 3 minutes, washed with PBS, centrifuged again before being resuspended in 30μl buffer and kept on ice.

5x10^5^ cells obtained from the exudates of the dermal techniques or from lysed whole blood were plated in a separate 96 well plate. The plate was centrifuged at 700g for 3 minutes and the cells resuspended in 30μl buffer.

Plates were then placed in an incubator for 15 minutes at 37°C. Subsequently the BioParticles were mixed with the cells using a multi-channel pipette. At time 0, the first of the two wells was aspirated and mixed with 60μl of 1% paraformaldehyde (Fisher Scientific UK) on ice, in a third 96-well plate, to prevent any phagocytosis from occurring (negative control). The plate was then incubated for 15 minutes at 37°C. After 15 minutes the second well was aspirated and mixed with 60μl of 1% formaldehyde on ice to stop phagocytosis.

The plate was centrifuged at 700g for 3 minutes; the cell pellet was washed in PBS, centrifuged again before being resuspended in 150μl of 0.5% formaldehyde on ice. The samples were analysed with the LSR fortessa, within 2 hours of completing the assay. Immediately prior to being run through the flow cytometer, the samples were quenched with 150μl 0.4mg/ml Trypan Blue (Sigma UK).

**Fig 3:**
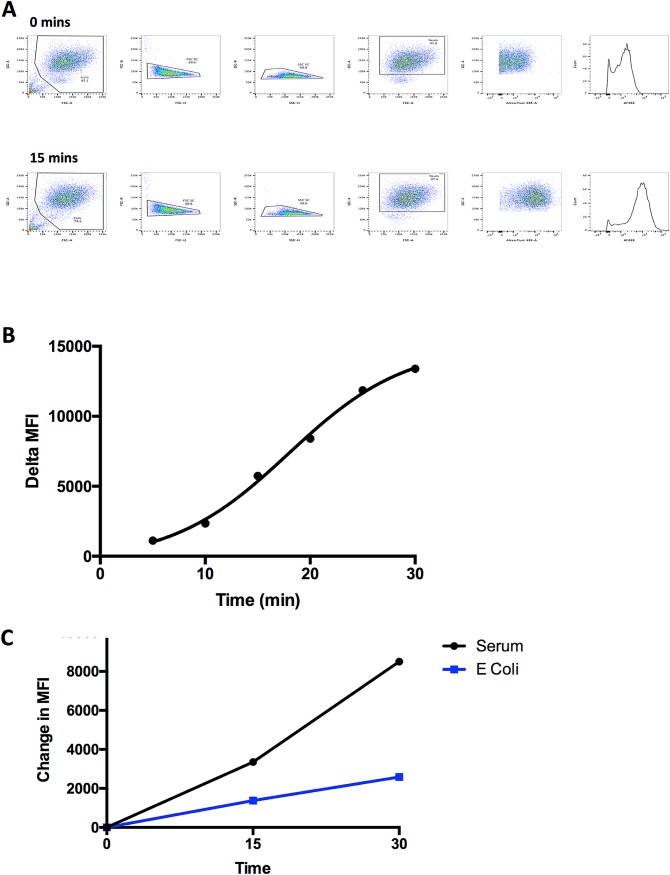
**Phagocytosis Assay** (A) Paired representative examples of flow cytometry gating strategy for phagocytosis assay using AF488-labelled *E*. *coli* as described in Methods. Exclusion of debris followed by exclusion of doublets. Selection of neutrophils using FSC/SSC. MFI calculated on single neutrophils. (B) Time-course of ECC neutrophil phagocytosis of AF488 *E*. *coli* particles at 5-minute intervals. Gated as in 3A. Expressed as change in MFI from baseline (time 0). (C) 5x10^6^ UVKEc in 50% autologous serum introduced into x2 ECC chambers and x2 chambers used as control. Phagocytosis assay performed after 4 hours of *in vivo* incubation.

The results were expressed as the change in MFI between the 0 minute and 15 minute samples (**Fig 3A**). The 15-minute time-point was selected after first performing a time-course using neutrophils from the ECC model. This demonstrated a sigmoidal curve (4PL nonlinear regression R^2^ = 0.9967) and the 15-minute time-point was selected as it was on the linear part of the curve (**Fig 3B**). We found that neutrophils that had previously encountered UVKEc had significantly reduced phagocytic capacity, presumably due to previous phagocytosis (**Fig 3C**).

### Statistics

Differences between fresh WB and the four models of neutrophil priming were tested for using unpaired, one-way ANOVA with Dunnett’s multiple comparisons test (with fresh WB set as the control). The difference in mean values between the 4-hour un-stimulated WB and 4-hour WB stimulated with LPS was analysed with paired *t* tests. Statistical analysis was performed in Prism 6 (GraphPad Software). Statistical significance was set at P<0.05.

## Results

### Cell counts

**Fig 4:**
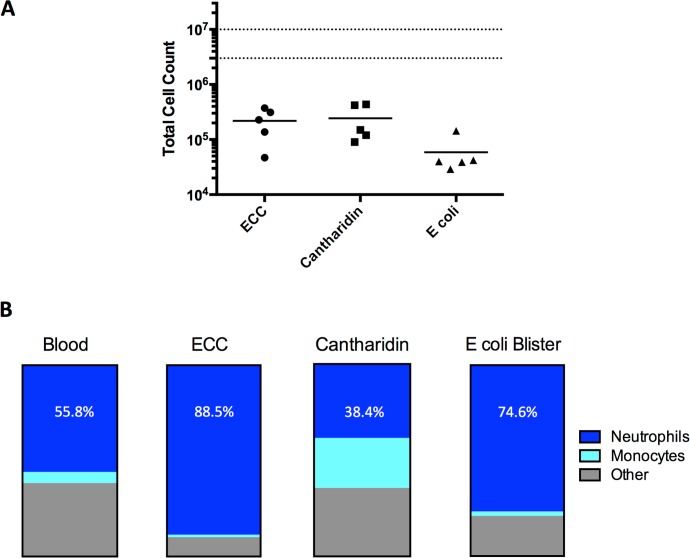
**Cell Count and Composition in the Four Models** (A) Total live cell count in each dermal model: ECC (4 hours, 4 chambers pooled), cantharidin (24 hours) and UVKEc-blisters (4 hours). Mean displayed (n = 5/group). Dotted lines represent normal range of number of leucocytes in 1ml of human blood (3-10x10^6^/ml). (B) Percentage of total live single cells making up blister samples split into neutrophils, total monocytes and other cells as per gating strategy in Fig 2. Mean displayed (n = 5/group).

The total number of cells from each of the dermal models was between 5x10^5^ to 1x10^6^ per volunteer (**Fig 4A**). There was no significant difference between the number of cells harvested from the 3 dermal models.

### Cell composition

The different techniques yielded significantly different cell populations (**Fig 4B**). Those from the ECC and UVKEc-blisters were comprised predominantly of neutrophils, whilst cantharidin blisters had a much higher proportion of monocytes.

### Neutrophil cell-surface markers

**Fig 5:**
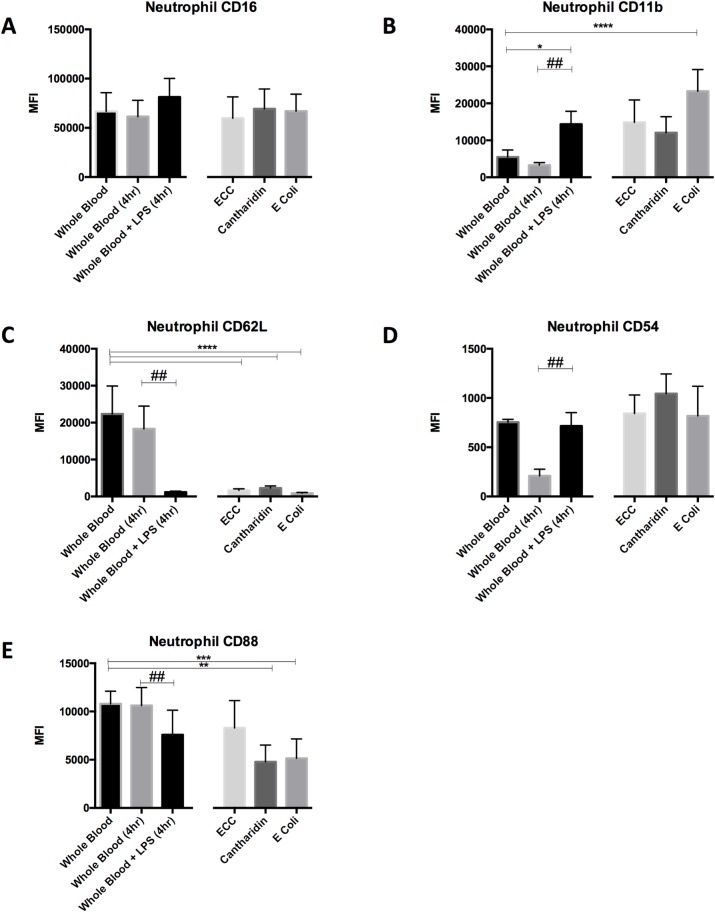
Neutrophil Cell-Surface Markers MFI of neutrophil cell-surface markers compared across the different techniques. Mean +SD displayed (n = 5/group). One-way ANOVA with Dunnett’s multiple comparisons test performed across the groups (excluding 4-Hr WB): *p<0.5, **p<0.01, ***p<0.001. ****p<0.0001. Paired *t* test performed between 4-Hr WB and 4-Hr WB + LPS: ^##^p<0.01 ^###^p<0.001.

Neutrophils were assessed for phenotypic activation using the panel of cell-surface markers described in Methods 2.7. MFI of the activation markers was compared across the different techniques. Although the trend of activation relative to fresh WB was similar in all 4 models of neutrophil priming with elevated CD11b and CD54 and reduced CD62L and CD88. However the degree of activation differed according to model used (**Fig 5**).

Mean MFI of CD11b was elevated in all four models, however only reached statistical significance versus fresh WB in the UVKEc-blister model (p<0.0001) and the LPS-stimulation model (p<0.05 vs fresh WB and p = 0.0035 vs 4-Hr WB). Changes in CD54 were less marked, with a trend towards increased expression in all dermal models vs fresh WB, and a significant increase in expression in the LPS-stimulation model versus 4-Hr WB (p = 0.001). CD62L was markedly and statistically significantly reduced in all four models versus fresh WB (p<0.0001). CD88 was also supressed in all four models, reaching statistical significance in the cantharidin (p<0.001) and UVKEc models (p<0.01) versus fresh WB and in the LPS model versus 4-Hr WB (p = 0.0057). There were no statistically significant changes in CD16 expression in any of the models.

Taken together, these data suggest that although the pattern of cell-surface markers is similar between all 4 models, neutrophils from the UVKEc model are the most activated.

### Neutrophil phagocytosis

**Fig 6:**
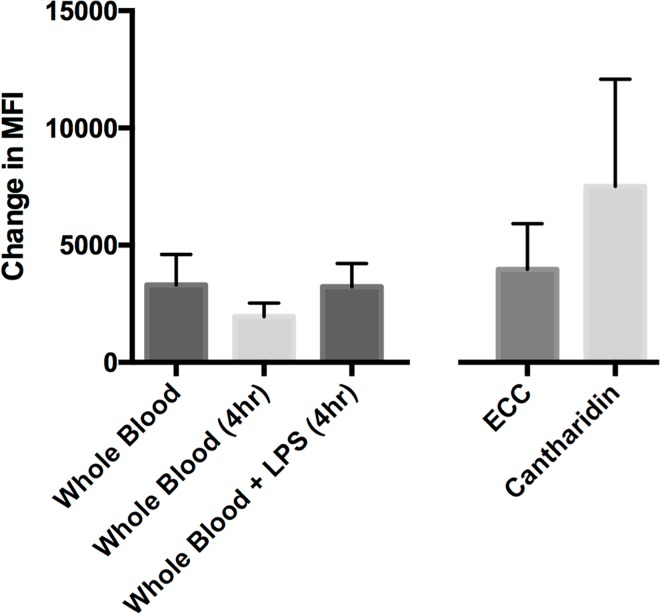
Neutrophil Phagocytosis All cells gated as shown in Fig 3A to give single neutrophils as defined by FSC & SSC. Results expressed as change in MFI from time 0 to 15-minute time-point. Mean +SD displayed (n = 5/group).

There was no significant difference in the phagocytic capacity of neutrophils between the three models of neutrophils priming relative to each other and fresh WB, however there was a trend towards those from the cantharidin blister having the greatest phagocytic capacity (**Fig 6**). Compared to un-stimulated blood incubated for 4 hours, blood stimulated by LPS had a higher phagocytic capacity (mean MFI 3236 v 1951), but this did not reach statistical significance.

## Discussion

### Neutrophil Activation

This is the first comparison of the activation status of primed human neutrophils obtained from three different dermal models of acute inflammation and *ex vivo* stimulation of WB with LPS.

The pattern of neutrophil cell-surface marker expression relative to circulating neutrophils was remarkably similar across all four models namely; reduced CD62L and CD88, elevated CD11b and CD54 and unchanged CD16. The most marked and consistent change across all models was the reduction in CD62L expression. This was anticipated, given that both the process of endothelial trans-migration (dermal models), and stimulation with LPS, are known to result in shedding of CD62L [18,19]. Shedding of CD62L plays a key role in regulating neutrophil migration as the resulting soluble ectodomain has a local anti-inflammatory effect [18].

The reduction in CD88 (C5a receptor) expression was also consistent, but more variable. Previous studies have shown that neutrophil priming, by stimulation with TNFα [20] or by non-inflammatory trans-migration to the lung [17], is associated with down-regulation of CD88. This was most marked in the cantharidin model–possibly due to the later time-point employed.

CD11b (Integrin alpha M) is part of the Mac-1 integrin that mediates adherence to the endothelium. Its expression is well-recognised marker of neutrophil activation [21]. Our data showed that this was elevated in all 4 models, but particularly high in the UVKEc-blister model. This may correlate with the amount of pro-inflammatory cytokines generated in each model. TNFα levels in the UVKEc-blister model have be measured at 1000pg/ml [15], whereas the levels in the cantharidin blister (70pg/ml) [12] and skin-window models (approx. 10pg/ml) [14] are considerably less.

The trend towards increased CD54 (ICAM) expression, did not reach statistical significance in any of the dermal models but modest rises in its expression in trans-migrated neutrophils is in keeping with previous reports [17].

The functional significance of neutrophil priming has been extensively demonstrated previously [8,9]. Our data showed a clear trend towards increased phagocytosis in the cantharidin and LPS models, however, given the small number of subjects, this did not reach statistical significance.

The overall pattern of cell-surface marker modulation observed in all four models of neutrophil priming is in agreement with previously published data examining the activation status of neutrophils present in BAL fluid [17]. However, BAL is an invasive technique requiring the relevant clinical training and experience. On the other hand, the models that we employed are minimally invasive and can be performed safely without the specialist clinical training and facilities required for BAL. Given that the activation pattern of neutrophils from all models were similar, the selection of the most appropriate model for obtaining primed neutrophils should be based on other considerations. Therefore, the major features of each of the models will now be discussed.

### I.d. injection of UVKEc

We consider the i.d. injection of UVKEc to be the ‘gold standard’ inflammation model that we used. It elicits the cardinal clinical signs of inflammation including redness, heat, swelling and pain and at the 4-hour time-point peak levels of pro-inflammatory cytokines, including TNFα, IL-1β, IL-8 and IL-6 are observed [15]. The neutrophils trans-migrate the endothelium, are subject to the full inflammatory milieu and encounter bacteria. Consistent with this, the neutrophils appeared the most activated, with the highest expression of CD11b and suppression of CD88.

The model also allows the full time-course of inflammation to be investigated [15]. The negative aspects of this model are that the associated pain and swelling may not be well tolerated by some subjects (particularly unwell patients) and neutrophil exposure to UVKEc renders the phagocytosis assay uninterpretable.

### ECC and Cantharidin

Whereas the ECC model produces a cellular profile very similar to that of the i.d. UVKEc model at the same time-point (4 hours), the profile obtained from the cantharidin blisters is significantly different with a large number of monocytes and lymphocytes [12]. This difference reflects the stage of inflammation, with the 24-hour time-point used in cantharidin representing the monocyte-driven resolution phase.

Neither of these models result in the cardinal clinical signs of inflammation as both are driven by superficial, local tissue damage and activation of neutrophils in these models is not augmented by additional stimulation (e.g. LPS or bacteria).

Although the pattern of activation markers was very similar between the two models, the phagocytosis data suggests that the neutrophils from cantharidin are functionally more active. This may be a result of the infiltrating monocytes present in the cantharidin blister contributing to the inflammatory milieu by producing TNFα as well as other pro-inflammatory cytokines.

Both models have previously been used to investigate functional defects in neutrophils. For example, Tritto *et al* demonstrated neutrophil phagocytic dysfunction in patients with cirrhotic liver disease using a version of the ECC model [22] and Harbord *et al* used cantharidin blisters to investigate defects in neutrophil trafficking and chemotaxis in patients with Crohn’s disease [3].

A potential unique feature of the ECC model is the ability to modify the fluid that is introduced into the four chambers. For example, inflammatory stimulants such as LPS or UVKEc, or pharmacological agents may be added to investigate the effect of these on neutrophil activation *in vivo* without significant systemic absorption and side effects. In contrast, cantharidin cannot be modified, but is a far simpler model to use and is well tolerated by the majority of subjects. Given the cellular profile and time-point the model lends itself to investigating the resolution of inflammation and allows the study of monocytes as well as neutrophils. It is also worth considering that cantharidin acts as a protein phosphatase 1 [23] and 2A inhibitor [24]. This may have unforeseen downstream effects on the acute inflammatory response particular to this method of eliciting blisters and their cellular content.

### WB Stimulation with LPS

Although this model is represents the least physiological means of obtaining primed neutrophils, the data suggests a similar phenotypic picture to dermal models.

This model has several obvious advantages. Firstly, the number of cells that can be obtained is substantially higher, meaning more functional assays can be performed. Secondly, taking blood is straightforward, quick and well tolerated by subjects whereas all the other models are more complex and incompatible with acutely unwell patients. Thirdly, other inflammatory cells aside from neutrophils can be studied, and the release of pro-inflammatory cytokines can be used as a proxy read-out for immune function [16]. These features explain why the model has been used far more extensively than the dermal models to study neutrophils in the acutely unwell patients.

### Limitations

The study was limited by several factors. Firstly, there were a limited number of healthy subjects enrolled in each group [5]. Although in total, 27 subjects were involved in the study, recruitment was a challenge. Even to healthy volunteers, the dermal models are a relatively invasive process. The low numbers had a particular impact on the phagocytosis data, as clear trends between groups did not reach statistical significance. A wide range of potential assays is available for testing the function of neutrophils, both in terms of migration and in terms of phagocytosis and pathogen killing. These include studying respiratory burst, chemotaxis, chemokinesis and bacterial killing assays. We only employed a single assay of neutrophil function due to the low cell numbers obtained in the blister models. Had it been possible to include more subjects, other assays as mentioned above would have been of interest and may have elucidated subtle differences between the neutrophils in the models used.

## Conclusions

We have compared the activation and function of human neutrophils in 3 dermal models of acute inflammation and contrasted this with *ex vivo* stimulation of WB with LPS. Our data suggests that the degree of activation in each model is very similar and that all are valid means of obtaining primed neutrophils.

Given this, the choice of model for human studies should take into account the associated advantages and limitations of each. Factors such as cell yield and composition, ease of use and subject tolerability should be considered.

None of the dermal techniques are compatible with the environment of an acute hospital. Alongside this, the intradermal injection of *UVKEc* may trigger unforeseen complications in a population known to have significant immune dysfunction [25]. Therefore on balance, due to its high cell yields, ease of performance and tolerability to patients, *ex vivo* stimulation of WB with LPS is the best option for studying activated neutrophils in acutely unwell, hospitalised inpatients.
